# Medical Opinion and Movement

**Published:** 1912-01-06

**Authors:** 


### MEDICAL OPINION AND MOVEMENT.

### Test Meals.

Much doubt has been thrown by various clinicians lately upon the value of hydrochloric acid estimations and similar chemical diagnosis of gastric lesions. The more optimistic conclusions of Messrs. Panton and Tidy set forth in the *Quarterly Journal of Medicine* are interesting, because they are founded on the consideration of 331 consecutive unselected test meals analysed by them in the London Hospital. They are careful to point out the sometimes overlooked truth that the diagnosis and treatment of gastric cases can never depend on test meals alone. But they do find that the simple methods of analysis which they employ yield very reliable information when taken in conjunction with a careful anamnesis and with clinical observation. For their methods the original paper should be consulted; their deductions can be briefly summarised. In normal stomachs Günzberg's reaction is positive, free HCl varies between 0.08 and 0.12 per cent., and acidity between 40 and 50. In carcinoma of the stomach Günzberg's reaction is nearly always negative (*i.e.* free HCl is absent); and the average total acidity is 26. Some dimethyl acidity is often present. When carcinoma is grafted on a simple ulcer, free HCl may be present even above the normal amount. In simple ulcer the average free HCl is 0.13, and the total acidity 58; and in duodenal ulcer the figures are even higher. Free HCl is usually absent in achylia gastrica, alcoholic chronic gastritis, severe anæmia, chronic pulmonary tuberculosis, and some forms of chronic dyspepsia.

### An Experiment in the Treatment of Diabetes.

In the *American Journal of the Medical Sciences* Dr. Sewall gives an account of a line of treatment which he was induced to try on theoretical grounds for a case of diabetes in a child, and subsequently in other patients. At seven years of age a girl was found to have a glycosuria, which was at first manifest only in the morning urine and was amenable to regulation of the diet. Later it became constant and unmanageable, and in despair the author pursued a new line of treatment, with results which for a time were most encouraging. Thereupon he began to treat adult patients in the same way with some success; but unfortunately the child patient, after doing well for a time, relapsed, and is now in a state of acetonuria. Twelve patients in all were under observation, and the author feels convinced that in a certain proportion of diabetics beyond middle age the metabolism and general symptoms can be improved, and the sugar removed temporarily at least from the urine, by the administration of an infusion of lean meat acidulated with hydrochloric acid. In the youthful patient neither beef infusion nor pancreatic infusion was alone effective; but the two together were able, during some months, to achieve this result when all other diets failed to control the production of sugar.

### Albumen Reaction of Phthisical Sputum.

The above, as lately described by Roger, results from the following very simple technique. Dissolve the sputum in water and add a few drops of acetic acid; for greater distinctness, filter, add a little sodium chloride to the filtrate, and the albumen will be precipitated on heating. The sputum must be fairly recent, at any rate not decomposed. In a further article in the *Presse Médicale*, the above author and Valensi give statistics by which they claim to prove that although a single positive reaction does not mean a diagnosis of tubercle, yet a single negative one excludes it. The test is thus the converse of staining for the bacillus of Koch, and would obviously form an invaluable complementary measure, if its reliability could be established. In another number of the same journal, Lecalplain, who gives a case in which the albumen reaction was useful in the differential diagnosis from pulmonary syphilis, also claims complete significance of a negative result. To prove a negative is always a difficult procedure, but the great practical importance of being able to exclude definitely such a common and serious disease would seem to warrant careful investigation. A useful line to work on would seem to be the digestion of large quantities of non-albuminous sputum with antiformin, and examination of the centrifuged deposit by inoculation and also bacteriologically.

### The Cleft Palate Controversy.

A useful account of the diverging views held on this question is given in the *Practitioner* by Mr. F. W. Goyder, who epitomises the discussions on the subject at the Royal Society of Medicine and at the British Medical Association meeting. The protagonists of the dispute are Mr. Berry on one side, Mr. Lane on the other. The former favours the late median operation, the latter the early flap method. The author admits himself hitherto an adherent of the Lane school; yet he pronounces that until the adherents of the flap method produce a consecutive series of cases as complete and as successful as those of Mr. Berry, their opinions can have no weight and their position is untenable. Also that unless the results as regards speech are as good as those of the majority of the median operators, flap methods will have to be abolished or remodelled; and that unless the mortality of the operation in infancy is lowered, early operations will have to be abandoned. Nevertheless he hopes that in a few years, when the children operated on by the flap method fairly recently begin to grow up, it will be shown that the flap method, properly carried out, yields the better final result; and he speaks for several surgeons besides himself when he promises, when the time arrives, to publish these remote results, whether favourable or the reverse, in order to help the final settlement of the question. The arguments for and against the two operations are marshalled with impartiality, and the essence of the controversy is clearly defined.

### A New Organism of Chronic Dysentery.

In the *Journal of the Royal Army Medical Corps*, Major W. S. Harrison describes a case of chronic dysentery following an acute attack which originated in Morocco. The clinical course of the case was quite typical of a fairly severe infection; the initial attack had been acute, and in the subsequent chronic stage the general health was much deteriorated. From the stools a bacillus was isolated in almost pure culture, having characters which seem to show that it has not been hitherto described. Bacillus coli was conspicuously absent, and Flexner's bacillus was found only once in thirty colonisations. The patient's serum failed to agglutinate laboratory strains of Flexner's and Shiga's and paratyphoid bacilli; but it agglutinated the bacillus isolated from the stools, in a dilution of 1 in 60 in an hour. No blood cells or amœbæ were found in the stools, which were watery and very offensive. The patient was carefully dieted, and the colon was regularly washed out with saline and quinine solutions. In addition he received weekly doses of 100 million of the bacillus which was present in such numbers in the stools. Under this treatment he soon got quite well, and in three weeks the unknown bacillus disappeared and its place was taken by bacillus coli. It seems evident that this bacillus must have been the cause of the dysentery; a long account of its cultural characteristics and of its effect upon animals is given.

### Hydatid Mole and Chorioepithelioma.

The close association which exists between these two uncommon lesions has been the subject of repeated comment within recent years. In the *American Journal of Obstetrics and Gynæcology* are two papers which emphasise once more the connections of these mysterious puerperal abnormalities. Dr. Vineberg reports four most interesting cases. In two of them true hydatid mole (*i.e.* without any trace of fœtus) was associated with eclampsia, a combination of which but three examples appear to have been previously put on record. In one of these cases chorioepithelioma developed subsequently. The other two cases were instances of chorioepithelioma following hydatid mole. In one case, in which hydatid mole and chorioepithelioma coexisted, a total hysterectomy was performed, and the eclamptic symptoms only appeared after the operation. Since there was no trace of fœtus, and the uterus itself was entirely removed, it is difficult to reconcile this case with the theory that eclampsia is due to the fœtus. The other paper is by Dr. Frank, who points out that in very rare cases of hydatid mole metastases occur, and the hydatid growth is itself truly malignant. He lays stress on the difficulty of giving a prognosis in cases of chorioepithelioma, on the fact that partial removal may suffice to bring about cure, and on the tendency of metastases to regress spontaneously. He concludes by remarking on the difficulty of deciding upon a safe but not over radical treatment.

### Warmed Ether-Vapour.

One of the most recent American "notions" for improving the technique of ether administration is to deliver the vapour warmed, instead of cold. Davis and Gwathmey have strongly advocated this procedure, and have attributed to the chilly vapour produced by the evaporation of ether on a gauze mask some share in the causation of pulmonary complications after anæsthesia. In the *Interstate Medical Journal* Professor Seelig waxes somewhat scornful over the complicated apparatus necessary to do this, and over the futility of the achievement when it is done. He points out that the specific heat of vapours is extremely low, which connotes that they are very rapidly and easily warmed, and just as rapidly and easily cooled. Ether vapour cannot possibly be heated to more than 97° F. unless it is under pressure; and when it is thus warmed under pressure it at once cools when the pressure is released and the vapour permitted to expand. The author has conducted experiments, both *in vitro* and on animals, and with both warmed and cooled ether vapour, and is led to conclude that the bronchial air is not lowered in temperature, despite the fact that ether vapour chilled by evaporation is inhaled. The warm nasal, oral, pharyngeal and tracheal passages are fully competent to warm the inspired air and ether, and elaborate apparatus to deliver the latter already warm is, in his opinion, a mere waste of time, and a quite impractical complication in technique.

### Essential Renal Hæmaturia.

This baffling condition, which has hitherto attracted much less attention in this country than in Germany, is the subject of a communication by Dr. Hale White to the *Quarterly Journal of Medicine.* The condition is described as one characterised by frequent and severe hæmorrhage from one kidney with continuous or paroxysmal pain, yet with no discoverable cause, either macroscopically or microscopically. Care must be taken to exclude cases of nervous angioma of the papillæ, which is quite a distinct lesion. This puzzling syndrome is, not unnaturally, commonly diagnosed as calculus or tuberculosis or new growth, notwithstanding the negative evidence of cystoscopy, *x*-ray examination, bacterial urinalysis, etc. It is doubtless not so very rare in Britain as the paucity of the literature would indicate, since Dr. White has collected five such cases from the practice of Guy's Hospital in the last twelve years. In reporting this series, he has fortunately been able to follow up each patient, and it appears that each one of them has done well after exploration, and is cured of a very troublesome source of invalidity. The letters of the patients all show that they have been restored to perfect health, except that one patient still passes blood at intervals, though he has been free now for eighteen months on end. In one case the kidney was excised (and found to be quite normal); in the other four exploration was done, with nephrorraphy as well in one patient.

